# Alcohol and Cardiovascular Disease—Modulation of Vascular Cell Function

**DOI:** 10.3390/nu4040297

**Published:** 2012-04-19

**Authors:** Paul A. Cahill, Eileen M. Redmond

**Affiliations:** 1 School of Biotechnology, Dublin City University, Glasnevin, Dublin 9, Ireland; Email: paul.cahill@dcu.ie; 2 Department of Surgery, University of Rochester Medical Center, 601 Elmwood Ave., Rochester, NY 14642, USA

**Keywords:** alcohol, ethanol, cardiovascular, atherosclerosis, endothelial cell, vascular smooth muscle, signaling, mechanisms

## Abstract

Alcohol is a commonly used drug worldwide. Epidemiological studies have identified alcohol consumption as a factor that may either positively or negatively influence many diseases including cardiovascular disease, certain cancers and dementia. Often there seems to be a differential effect of various drinking patterns, with frequent moderate consumption of alcohol being salutary and binge drinking or chronic abuse being deleterious to one’s health. A better understanding of the cellular and molecular mechanisms mediating the many effects of alcohol consumption is beginning to emerge, as well as a clearer picture as to whether these effects are due to the direct actions of alcohol itself, or caused in part by its metabolites, e.g., acetaldehyde, or by incidental components present in the alcoholic beverage (e.g., polyphenols in red wine). This review will discuss evidence to date as to how alcohol (ethanol) might affect atherosclerosis that underlies cardiovascular and cerebrovascular disease, and the putative mechanisms involved, focusing on vascular endothelial and smooth muscle cell effects.

## 1. Introduction/Epidemiological Evidence and Animal Study Evidence

Epidemiologic studies that examine patterns of health and disease and associated factors in a population, point to a complex association between alcohol consumption and cardiovascular disease. With few exceptions, studies from several countries demonstrate a 20–40% lower cardiovascular disease incidence among drinkers of alcoholic beverages compared with non-drinkers. But what level of consumption is most beneficial and does drinking pattern and/or beverage choice matter? The general consensus currently is that compared with abstinence, frequent moderate consumption of alcohol is associated with the lowest risk for coronary heart disease incidence and mortality [1,2]. For example, 1–2 drinks per day is a negative risk factor for atherosclerosis and its clinical sequelae myocardial infarction and ischemic stroke [3]. On the other hand binge or heavy episodic drinking, defined in the USA as consuming 5 or more drinks in a relatively short time period, is associated with increased cardiovascular disease and associated mortality [4,5,6]. In terms of beverage choice, some studies report equal effects of wine, beer or liquor on cardiovascular disease risk [3,7], while other studies support a preferential red wine protective effect, attributable to both the alcohol (ethanol) and the polyphenolic antioxidant content, in particular resveratrol [8,9]. Moreover, some white wines are also reportedly cardioprotective, believed to be due to the presence of tyrosol and hydroxytyrosol [10,11,12].

In addition to epidemiological studies, moderate alcohol consumption has been shown to prevent the development and progression of atherosclerosis in a variety of animal/mouse models (C57 hyperlipidemic mice) [13,14], (LDL receptor knockout (LDLR −/−) mice) [15], (ligation injury) [16]. A recent study in mice demonstrated differential effects of daily moderate and 2-day binge ethanol drinking (good and bad, respectively) on atherosclerotic plaque development, body weight gain and low density lipoprotein (LDL)-cholesterol levels in ligated Apolipoprotein E (ApoE) knockout (−/−) mice, in apparent agreement with epidemiologic findings in humans [17,18]. Conversely, Bentzon* et al.* reported that neither ethanol nor red wine affected established atherosclerotic plaques in ApoE −/− mice [19]. Similarly, beer consumption did not alter the development of atherosclerosis in mice [20]. Reasons for these conflicting results likely include different experimental variables such as the strain of mouse [21], type of model (e.g., ApoE −/− *vs*. LDLR −/− *vs*. ligation injury *vs*. endothelial denudation) and diet (e.g., differences in fat and cholate content [22]) used, as well as the stage of lesion development assessed. Mouse models of accelerated atherosclerosis, while not perfectly replicating the complexity of the human disease, nevertheless remain useful in the investigation of the pathogenesis of atherosclerosis as well as understanding how alcohol consumption may affect it, as long as care is taken in extrapolating results to humans. Of note in the alcohol research field is the variety of units used to describe alcohol treatment and or levels achieved, especially in animal studies; g/kg, mg, g%, millimolar, *etc.* This sometimes makes it difficult to accurately compare results from different studies as well as to relate the experimental conditions to levels of consumption in humans. For reference, in the USA 1 unit of alcohol (*i.e.*, 12 oz beer, 5 oz of wine or 1.5 oz of liquor) contains 14 g of pure alcohol and would give rise to a blood alcohol content in the range 0.02–0.03%, equivalent to approximately 5 mM. (The blood concentration considered legally impairing is 0.08% v/v, which is about 17 mM). Ethanol (EtOH) is the type of alcohol found in alcoholic beverages and is used interchangeably with alcohol in this article.

## 2. Atherosclerosis

Atherosclerosis, a chronic inflammatory condition in which the artery wall thickens as a result of the accumulation of cholesterol, macrophages and smooth muscle cells (SMC), ultimately restricting blood flow through the artery, is the main pathologic condition underlying coronary artery and cerebrovascular disease leading to heart attack and stroke, respectively. In the pathogenesis of atherosclerosis (reviewed in [23,24,25,26]), increases in plasma low density lipoprotein (LDL) leads to a proportional increase in the entry of cholesterol laden LDL particles into the arterial wall across a “compromised” endothelial monolayer, where it accumulates. Once there, it can become oxidized, possibly by free radical production from adjacent endothelium, smooth muscle cells or an isolated macrophage [27,28,29,30]. Oxidized LDL has numerous effects on a variety of cells, many of which are believed to cumulatively exacerbate atherothrombosis ([31] for review). These include promotion of monocyte adhesion and infiltration to the intima by causing production of monocyte chemotactic protein-1 (MCP-1) by endothelium and expression by endothelium of monocyte-binding proteins including intercellular adhesion molecule-1 (ICAM-1), foam cell formation following uptake of oxidized LDL via scavenger receptors (SR-A type I and II and CD36), and stimulation of the migration of medial SMC into the intima where they proliferate in response to growth factors such as platelet derived growth factor (PDGF) [23,25]. In the intima, SMC produce extracellular matrix molecules including collagen and elastin. The most common clinical complication of atherosclerosis occurs upon plaque rupture which allows blood components to come into contact with plaque lipids and tissue factor, resulting in thrombus formation. It is obvious then that there are many steps in atherogenesis that, were they affected by alcohol, might affect plaque development and subsequently myocardial infarction or stroke. Recent articles have reviewed alcohol’s effects on lipids, fibrinolysis and inflammation [32,33]. As endothelial dysfunction, together with the migration and proliferation of normally quiescent SMC plays a fundamental role in atherogenesis, we will focus in this review on alcohol effects on these two cell types. 

It should be noted that with respect to alcohol and the vasculature in general, the majority of studies have focused on arterial effects, as these are most relevant to cardiovascular disease. However, some studies have addressed the relationship between alcohol consumption and risk of venous thrombosis and venous thromboembolism (VTE). Pahor *et al.* reported that low to moderate alcohol consumption was associated with a decreased risk of deep vein thrombosis and pulmonary embolism in a cohort of people aged 68 years or older [34]. Pomp *et al.* came to a similar conclusion finding that alcohol consumption was associated with a reduced risk of venous thrombosis, an effect more pronounced in women, and one which may be mediated by decreased fibrinogen levels [35]. A more recent study attempted to dissect out the effect of different types of alcoholic beverage and drinking pattern and found that whereas liquor consumption and binge drinking was associated with an increased risk of VTE, wine drinking was associated with a reduced risk [36]. Thus, similar to alcohol’s effect on cardiovascular disease, its effect on VTE may depend on the pattern of consumption, as well as the type of alcoholic beverage consumed.

## 3. Alcohol and Nitric Oxide (NO)

Nitric oxide (NO), also known as endothelium-derived relaxing factor (EDRF), is a key regulatory signaling molecule in the vasculature [37]. It is synthesized by the heme-containing, calcium and calmodulin-dependent enzyme nitric oxide synthase in endothelial cells (eNOS) from L-arginine in a reaction that produces stoichiometric amounts of L-citrulline [37,38]. Activation of NOS and release of NO results in stimulation of a soluble guanylyl cyclase leading to a profound increase in intracellular cyclic guanosine monophosphate (cGMP) levels within most target cells [38]. NO has a wide range of actions important in maintaining vascular homeostasis. In addition to causing vasodilation, it has antiproliferative, antioxidant and anti-inflammatory properties that inhibit atherogenesis ([39] for recent review). Common risk factors for atherosclerosis such as hypercholesterolemia, hypertension, smoking and diabetes mellitus are associated with reduced NO in the arterial wall. Because of these findings, numerous therapies have been investigated based on enhancing NO release, thereby reversing endothelial dysfunction and preventing atherogenesis. 

Researchers have wondered whether moderate alcohol consumption mediates some of its cardioprotective effects by stimulating NO, and conversely, whether binge drinking diminishes NO availability. Initial studies in cultured bovine aortic endothelial cells (BAEC) and human umbilical vein endothelial cells (HUVEC) reported that ethanol treatment increased NO production by enhancing NOS activity [40,41,42]. Abou-Agag *et al.* fed rats alcohol at moderate levels for 8 weeks before evaluating NO production and post-ischemic myocardial function and vascular relaxation *ex vivo* [43]. Their results indicated that moderate alcohol consumption increased the expression of eNOS protein in the vasculature and NO metabolites in the blood, an effect associated with enhanced postischemic myocardial systolic and diastolic function as well as attenuated ischemia-induced coronary vascular resistance [43]. Kleinhenz* et al.* published similar findings looking at aortic NOS expression, NO production and relaxation to acetylcholine in alcohol fed rats [44]. There is some evidence for gender differences in NOS-dependent vascular responsiveness to alcohol consumption, but this seems to involve inducible isoform NOS (iNOS) [45]. Of interest, male Fisher rats treated chronically (12 weeks) with high “abuse” levels (4 g/kg) of alcohol daily via orogastric tube developed hypertension, impaired vascular relaxation, reduced vascular eNOS expression, and evidence of increased vascular oxidative stress [46,47]. It is apparent that the dose and length of EtOH exposure, and cell type are the main factors affecting EtOH effects on NO production. Polikandriotis *et al.* reported that chronic ethanol ingestion increased NO release from pulmonary endothelial cells by a mechanism involving phosphatidylinositol 3-kinase (PI3K)-mediated increases in eNOS expression and increases in protein-protein interactions between eNOS and hsp90 [48]. In female rats, ethanol increased myocardial expressions of eNOS and its upstream regulators, PI3K and Akt (also known as protein kinase B), and plasma endotoxin and nitrite/nitrate were increased by ethanol [49].

Human studies show similar results; e.g., ethanol and red wine consumption acutely increased the production of NO in healthy subjects [50]. However, in a recent study comparing the effect of water, red wine, beer and vodka in healthy young subjects Huang *et al.* found that only red wine affected endothelial function (determined by flow-mediated vasodilation) and significantly increased plasma levels of nitric oxide [51]. Overall however, most studies in cells, animals and human subjects show a beneficial effect of moderate ethanol treatment on NO. A recent paper sheds more light on the molecular mechanisms that may be involved [52]. Using human aortic endothelial cells (HAEC), they showed that rapid activation of mitochondrial aldehyde dehydrogenase 2 (ALDH2), a key enzyme in ethanol metabolism, was involved in ethanol-induced eNOS activation by preventing reactive oxygen species (ROS) accumulation. ROS are implicated in atherosclerosis via inactivation of NO. Moreover, ethanol-induced ALDH2 activation was dependent on its acetylation modification by NAD-dependent deacetylase sirtuin-3 (SIRT3) inactivation, the latter due to a reduced NAD^+^/NADH ratio in mitochondria [52]. Kuhlmann *et al.* reported that EtOH directly activates Ca^2+^-activated potassium channels in HUVEC, leading to increased production of NO (at EtOH concentrations of 10–50 mM) [53]. Higher concentrations of EtOH (100 and 150 mM) significantly reduced NO synthesis [53]. Taken together, these studies are supportive of a protective effect of moderate alcohol and a deleterious effect of alcohol abuse via differential modulation of NOS activity and NO production in the vasculature. Changes in NO levels may partly explain the variable dilatory and constrictive effects reported for alcohol in different vascular beds [54,55,56,57]. Other mechanisms likely include ethanol-induced changes in the levels of the vasodilator prostacyclin (PGI_2_) [58] or the potent vasoconstrictor endothelin-1 [59], as well as changes in intracellular Ca^2+^ [54,60,61] and Mg^2+^ levels [62]. With respect to vasoreactivity and alcohol, once again there is evidence of opposite effects of low-moderate (vasodilatory) *vs*. high consumption (vasoconstrictive) [62].

## 4. Endothelial Progenitor Cells

Evidence indicates that the injured endothelial monolayer may be regenerated by circulating bone marrow-derived endothelial progenitor cells (EPC), which accelerate re-endothelialization and protect against the initiation and progression of atherosclerosis [63,64]. Higher circulating levels of progenitor cells reflect greater repair capacity and have been shown to reduce the progression of atherosclerosis [65]. Of interest, a handful of recent studies suggest that alcohol consumption may increase the number of circulating EPC. Moderate red wine consumption improved neovascularization and blood flow recovery after ischemia in hypercholesterolemic mice and had a positive effect on EPC number and functional activity [66]. Huang* et al.* reported that red wine consumption by healthy subjects enhanced circulating EPC levels and improved EPC functions by modifying NO bioavailability [51]. In a mouse model of atherosclerosis (*i.e.*, angiotensin II infusion in ApoE −/−) Gil-Bernabe *et al.* demonstrated that the low-dose ethanol treatment group had fewer atheromatous lesions, and increased secretion of stromal cell-derived factor-1 (SDF-1) with subsequent enhanced mobilization of progenitor cells, compared to the no alcohol controls [67]. Collectively, these studies support a modulatory effect of ethanol and/or polyphenols on EPC that may be anti-atherogenic.

## 5. Alcohol and Reactive Oxygen Species (ROS). Alcohol: Prooxidant or Antioxidant?

Increased production of reactive oxygen species (ROS) contributes to mechanisms of vascular/endothelial dysfunction and atherosclerosis [68,69]. Oxidative stress is mainly caused by an imbalance between the activity of endogenous pro-oxidative enzymes (such as NADPH oxidase, xanthine oxidase, or the mitochondrial respiratory chain) and anti-oxidative enzymes (such as superoxide dismutase, glutathione peroxidase, heme oxygenase, thioredoxin peroxidase/peroxiredoxin, catalase, and paraoxonase) in favor of the former. ROS may play a role in mediating alcohol’s various effects, particularly in relation to its vasorelaxant effect and its protective effect against ischemia reperfusion injury.

Rocha *et al.* recently reported that scavenging of superoxide anion (O_2_^−^) and hydrogen peroxide (H_2_O_2_), both important ROS in the vascular wall, prevented ethanol-induced aortic relaxation, suggesting that the response was mediated, in part, by oxidative stress [70]. Their data further suggested that ROS generation triggers the activation of the NO–cGMP pathway, which in turn increases NO generation and relaxation [70]. These findings are supportive of a redox-sensitive and NO-dependent signaling mechanism underlying low to moderate ethanol-induced vascular relaxation. On the other hand, chronic alcohol abuse is associated with hypertension in animals and humans [45] and pharmacological (high) doses of ethanol induce vasoconstriction in aortic ring studies via redox-sensitive and cyclooxygenase-dependent signaling [60]. 

Oxidative stress induced by ROS plays an important role in the pathogenesis of ischemia/reperfusion injury. The regular moderate consumption of alcoholic beverages is believed to protect against ischemia-induced myocardial injury, in a manner similar to ischemic preconditioning (IPC), possibly by affecting the prooxidant/antioxidant balance. This area has been thoroughly reviewed previously [71,72].

## 6. Ethanol and Endothelial Proliferation, Migration and Angiogenesis

Endothelial cell (EC) dysfunction and/or loss, in response to a wide range of injurious stimuli (e.g., high levels of LDL, smoking, low shear stress, iatrogenic manipulation), resulting in compromise of the protective endothelial barrier is acknowledged as a key initiating step in atherogenesis [23]. Stimulation of EC proliferation and/or migration in this context by alcohol might therefore be perceived as cardiovascular protective. Indeed, ethanol treatment of cultured EC at levels consistent with moderate consumption enhanced their proliferation, migration, and network formation on Matrigel (an index of angiogenic activity) [73]. This pro-angiogenic effect of ethanol was mediated via stimulation of a novel Notch-Angiopoietin 1 signaling pathway in these cells and this study provided the first evidence of the Notch pathway as a novel mechanistic target for ethanol [73]. The Notch pathway is a signaling mechanism important in vascular development, playing a key role in vascular cell fate decisions [74,75]. Notch receptors are expressed on both EC and SMC and their role in adult vascular physiology has being unveiled by researchers over the last 15 years. Of note, the function of the Notch pathway in EC may be dependent on the endothelial type, and activation of different Notch receptors by different ligands may elicit opposing responses. For example, Notch signaling can either inhibit or stimulate EC proliferation and migration [73,76,77,78]. Similarly, Notch reportedly has both pro- [73,79] and anti-angiogenic [80,81,82] effects. 

Migration and proliferation of endothelial cells is central to angiogenesis, *i.e.*, the growth of new capillary blood vessels. Diseases characterized by abnormal or excessive angiogenesis include cancer, psoriasis and rheumatoid arthritis. Diseases characterized by insufficient angiogenesis or vessel regression include Alzheimers disease, diabetes, stroke, ischemic heart disease and restenosis [83]. Thus, angiogenesis is beneficial in some clinical circumstances but maladaptive in other situations and control of angiogenesis therefore represents an area with rich therapeutic potential. With respect to cardiovascular disease specifically, the role of angiogenesis is complex as it can be beneficial or deleterious depending on the context; e.g., collateral vessel formation in response to ischemia *vs*. intraplaque neovasculature, respectively. Many advanced atherosclerotic lesions are vascularized, especially the “vulnerable” plaque regions [84], by a network of capillaries that arise from the adventitial vasa vasorum [85]. Plaque angiogenesis seems to characterize the inflammatory, more “active” plaque rather than the calcified, more “inactive” plaque and a positive association between neovessel density and plaque rupture has been reported [86].

In contrast to plaque angiogenesis, compensatory or collateral angiogenesis (e.g., coronary collaterals) is a physiological process in response to occlusion ischemia. Collaterals are induced over a period of several days-weeks and require tissue ischemia from existing vascular stenosis. The process results in the formation of mature vessels (“natural bypasses”) that can compensate for the loss of perfusion following myocardial infarction or stroke. Thus, the development of a collateral circulation plays an important role in protecting tissues from ischemic damage and its stimulation has emerged as one of the principal approaches to therapeutic angiogenesis [87]. Interestingly, clinical observations detail substantial differences in the extent of collateralization among patients with coronary artery disease, with some individuals demonstrating marked abundance and others showing nearly complete absence of these vessels [87,88]. Factors responsible for the presence or absence of collateral circulation are poorly understood but genetic and lifestyle factors, such as alcohol consumption, are likely to play a role. A relationship between alcohol and angiogenesis has previously been investigated. Radek *et al.* showed that acute ethanol exposure inhibited angiogenesis in a murine model of wound healing [89]. On the other hand, several groups report a stimulatory effect of ethanol on angiogenesis (particularly in relation to tumorigenesis) in a variety of *in vivo* and *in vitro* models [73,90,91,92,93,94]. The mechanisms involved included ethanol stimulation of angiogenic growth factors such as vascular endothelial growth factor (VEGF) [90,92], basic fibroblast growth factor (bFGF) [92,93], and transforming growth factor beta (TGFβ)1 [93], while Qian *et al.* provided evidence of a signaling pathway linking ethanol-induced changes in cell division control protein 42 homolog (Cdc42), H_2_O_2_, actin filaments and cell motility to *in vitro* angiogenesis [95]. Endothelial cell migration and proliferation are central to the process of new blood vessel formation and a biphasic effect of ethanol, whereby low dose ethanol (1–30 mM) stimulates and higher dose ethanol (30–100 mM) inhibits EC proliferation and migration has also been reported [53,96]. The role of NO in angiogenesis remains controversial with multiple lines of evidence for both pro-angiogenic and anti-angiogenic activity ([97] for review). As mentioned earlier, ethanol has been previously shown to modulate nitric oxide synthase (NOS) activity and NO production [40,41]. Of interest, in relation to coronary collateral formation, a recent study found an association between alcohol consumption and the presence of collaterals in patients with documented coronary artery disease [98].

## 7. Monocyte Chemotactic Protein-1

Monocyte chemotactic protein-1 (MCP-1) plays an important role in the recruitment and activation of monocytes and thus in the development of atherosclerosis. In response to several atherogenic stimulants such as oxidized LDL, platelet derived growth factor (PDGF) and interleukin-1β (IL-1β), MCP-1 is induced in endothelial cells, smooth muscle cells and monocytes [99]. MCP-1 mediates its biological activity mainly through interaction with an MCP-1 receptor, CCR2, (also known as C-C chemokine receptor) on the surface of its target cells which include monocytes. This receptor belongs to the superfamily of G protein-coupled receptors with seven transmembrane domains. The important role of CCR2 in atherogenesis has been demonstrated in studies using gene knockout animal models; there was a marked decrease in atherosclerotic lesion formation in apo-E-null mice that lacked CCR2 [100] and increased CCR2 expression is evident in patients with hypercholesterolemia [101]. Several agents, including homocysteine and oxidized LDL, have been shown to affect CCR2 expression [102]. An alcohol effect on MCP-1 and/or its receptor would therefore be of clinical interest/relevance, and it has been investigated* in vitro* and *in vivo*. In HUVEC, Cullen* et al.* demonstrated that although EtOH had no effect on monocyte CCR2 binding activity, it inhibited IL-1β-stimulated endothelial MCP-1 expression by decreasing MCP-1 mRNA stability, binding of the transcription factors nuclear factor kappa B (NF-κB) and activator protein-1 (AP-1), and MCP-1 gene transcription [103]. These data suggest a possible mechanism whereby EtOH could block monocyte adhesion and subsequent recruitment to the sub endothelial space and thus inhibit atherogenesis. In apparent support of this hypothesis, moderate consumption of ethanol or de-alcoholized red wine over 3 weeks by healthy subjects resulted in a significant inhibitory effect on MCP-1-induced migration of monocytes *ex vivo* and* in vitro*, with no effect on MCP-1 receptor expression [104]. However, in another study in healthy volunteers comparing the effect of different alcoholic beverages containing more or less polyphenols, Blanco-Colio* et al.* found that only beverages with the highest polyphenol content (e.g., red wine) inhibited plasma MCP-1 concentrations [105]. Similarly, Vazquez-Agnell* et al.* reported an inhibition of MCP-1 following sparkling wine (medium level polyphenol content) consumption but not gin (no polyphenol content) [106]. Taken together, these studies support an effect of both ethanol and polyphenols in modulating MCP-1 expression. Whether the MCP-1 inhibitory effect of alcoholic beverages is due primarily to the polyphenol content rather than the ethanol, per se, requires further investigation.

Of note, the primary step in the metabolism of alcohol is its oxidation to acetaldehyde by the enzyme alcohol dehydrogenase (ADH). Acetaldehyde is rapidly converted to acetate by other enzymes and is eventually metabolized to carbon dioxide and water. Acetaldehyde itself has been shown to have multiple cardiovascular effects *in vivo*, including vasodilation, increased heart rate and decreased blood pressure [107]. *In vitro*, acetaldehyde increased monocyte adhesion to cultured EC and stimulated P-selectin and TNFα expression [108]. It is possible, particularly in binge drinking scenarios where elevated blood acetaldehyde levels have been reported [109], that some of the cardiovascular effects of alcohol consumption are due to a combination of the direct actions of ethanol itself in combination with effects due to its primary metabolite, acetaldehyde. The balance between ethanol and acetaldehyde levels may be an important factor to consider in the beneficial *vs*. deleterious effects of different drinking patterns [17]. 

## 8. Smooth Muscle Cell (SMC) Differentiation/Phenotypic Switching and Vascular Disease

In the adult, vascular smooth muscle cells (SMC) proliferate at an extremely low rate and their principal function is contraction and regulation of blood vessel diameter. Unlike either skeletal or cardiac muscle that are terminally differentiated, SMC within adult animals retain plasticity and can undergo reversible changes in phenotype in response to a variety of local environmental cues such as growth factors or mechanical forces [110]. An example of this plasticity can be seen in response to vascular injury when SMC dramatically increase their rate of cell proliferation, migration and synthetic capacity (including the production of extracellular matrix components). There is a strong body of evidence that this “phenotypic switching” or “differentiation” plays a major role in a number of human diseases, in particular vascular proliferative pathologies including atherosclerosis and restenosis [111,112]. 

Several groups have investigated the effect of alcohol on injury-induced vascular remodeling/intimal medial thickening that is driven predominately by a smooth muscle migratory and proliferative response. An ethanol-induced reduction in neointimal formation following balloon injury has been reported in rabbits and pigs [113,114,115]. This inhibition of intimal hyperplasia was observed following either local delivery of ethanol or alcohol feeding [113,114]. The preservation of arterial lumen diameter was achieved by decreasing neointimal proliferation in part by decreasing LDL oxidation in these animals. Emeson *et al.* demonstrated in mice fed a high fat diet that alcohol feeding not only inhibited the initial development of atherosclerotic lesions, but also inhibited the progression of existing lesions [14]. In a study using rabbit iliac arteries following balloon angioplasty, significant inhibition of SMC phenotype conversion from contractile to synthetic was observed following ethanol treatment that was indicative of an inhibition of SMC proliferation [113]. In the porcine balloon-overstretch model, perivascular administration of a single-dose ethanol reduced neointimal proliferation [116]. Moderate daily consumption of ethanol inhibited carotid intimal-medial thickening after ligation injury/flow reduction in mice [16,17], whereas 2 day/week binge alcohol consumption exacerbated it [17]. Moreover, in a retrospective cohort study involving 225 male patients, Niroomand *et al.* reported that alcohol intake (≥50 g alcohol/week) was associated with reduced restenosis after percutaneous transluminal coronary angioplasty (PTCA) and stent implantation in patients, and a lower rate of repeat angioplasty [117].

A number of studies employing isolated vascular SMC in culture confirm an anti-proliferative effect of alcohol in these cells. Ethanol dose-dependently inhibited serum-stimulated mitogenesis and proliferation in rat aortic SMC [118]. The activity of mitogen activated protein kinases (MAPKs), which play a key role in regulating SMC growth [119] was inhibited by ethanol in these cells [118]. A differential modulation of key cell cycle regulatory molecules by ethanol, including the induction of the cyclin-dependent kinase (Cdk) inhibitor p21^waf1/cip1^, and inhibition of cyclin A, has also been reported and may be the mechanism by which ethanol inhibits G1→S phase progression of the cell cycle and thus SMC proliferation [120]. Inhibition of SMC growth by ethanol has also been correlated with a reduction in fibroblast growth factor (FGF)-dependent MAPK activation [121]. Lochner* et al.* demonstrated that the stimulatory effect of postprandial plasma on SMC proliferation was reduced significantly by the concomitant ingestion of ethanol in healthy subjects [122]. Of note, as NO itself is a potent inhibitor of vascular smooth muscle cell proliferation [123,124] ethanol may also affect vascular SMC growth indirectly via its stimulatory effect on endothelial cell NO production. 

### Notch, Vascular Injury and SMC Proliferation

Notch receptors and downstream target genes (Hes, Hrt) are crucial in controlling adult vascular SMC growth, migration and apoptosis* in vitro* and* in vivo *[125,126,127]. Cyclic strain, due to pulsatile blood flow, modulates Notch receptor signaling and proliferation in vascular SMC [128]. Moreover, previous studies suggest that the expression of several Notch components, including receptors (Notch 1 and Notch 3), ligands (Jag1, Jag2) and target genes (Hrt-1 and Hrt-2) are altered after experimentally-induced vascular injury (balloon catheter denudation) [126,129,130] and in human atherosclerotic lesions [131]. Intimal hyperplasia after vascular injury was significantly decreased in Hrt 2 −/− mice [132] and SMC from Hrt 2 −/− mice revealed that these mutant cells proliferate at a reduced rate compared with wild-type cells while the over-expression of Hrt 1 or Hrt 2 in VSMC led to increased VSMC proliferation associated with reduced levels of the cyclin-dependent kinase inhibitors p21^waf1/cip1^ (Cdkn1a) or p27^kip1^ (Cdkn1b) [133]. Recently, Morrow* et al.* investigated whether modulation of Notch signaling may mediate alcohol’s inhibitory effect on SMC proliferation. They found that ethanol treatment selectively inhibited Notch 1 mRNA and decreased CBF-1/RBPjk promoter activity and Notch target gene expression, concomitant with inhibition of SMC proliferation* in vitro* [16]. Moreover, Notch 1 and HRT-1 expression induced after ligation injury, was inhibited by moderate alcohol feeding in mice [16]. Overexpression of constitutively active Notch 1 IC or hHRT-1 prevented the ethanol-induced inhibition of SMC proliferation [16]. Thus, it appears that ethanol inhibits SMC proliferation by inhibiting Notch signaling in these cells [16], effects opposite to that reported in EC [73]. It is conceivable, however, that stimulation of EC proliferation together with simultaneous inhibition of SMC by ethanol would be synergistically protective against atherogenesis. In any case, these data indicate that ethanol has an intriguing differential effect on Notch signaling and growth, depending on the cell type. Further investigation is warranted to understand the mechanisms mediating these differential effects.

In addition to proliferation, migration of SMC from the media to the intima is a prominent feature of atherogenesis. SMC migration requires degradation of the extracellular matrix (ECM) a process which involves the plasminogen/plasmin and matrix metalloproteinase (MMP) systems. Ethanol treatment inhibited basal and pulse pressure-induced migration of human SMC [134] by stimulating plasminogen activator inhibitor-1 (PAI-1) and concomitantly inhibiting MMP-2 and MMP-9 [135]. Analogously, moderate consumption of beer or alcoholic beverages in healthy subjects reduced MMP-2 plasma activity, with no effect on MMP-2 expression or antioxidant activity levels [136].

## 9. Molecular Mechanism of Action?

Clearly, numerous biochemical and physiological effects of ethanol have been described over the past few decades. It is somewhat surprising then that relatively little is yet known about precisely how ethanol acts mechanistically to produce its many effects, cardiovascular or otherwise. While an “ethanol receptor” has not yet been identified, more information re ethanol’s molecular targets is slowly emerging (reviewed in [137]). Moreover, ethanol may act on membrane proteins by disrupting protein-lipid interactions. One hypothesis is that if this occurred relatively specifically in lipid rafts (*i.e.*, membrane microdomains enriched in cholesterol and sphingomyelin) which are emerging as important players in signal transduction because of their ability to concentrate and assemble signaling molecules, the movement of proteins into or out of rafts and therefore signaling through several receptors types, could be modulated [138]. In support of this, recent evidence supports a role for lipid rafts in the actions of ethanol on macrophage activation and TLR4 signaling [139,140]. More precise knowledge of ethanol’s molecular targets and/or mechanisms of action should lead to the development of therapeutic agents that can mimic the beneficial effects of alcohol and/or block its deleterious and intoxicating effects. 

Investigation of microRNAs (miRNAs) as possible targets for ethanol has also begun, particularly in relation to alcohol abuse and toxicity (reviewed in [141]), and may yield new information with respect to alcohol’s pleiotropic cardiovascular effects. miRNAs are small, non-coding RNAs that regulate gene expression by binding to complementary sequences within target mRNAs. They have been shown to play critical roles in a broad array of fundamental biologic processes including regulation of the cell cycle and cell differentiation [141], and they have been implicated in vascular pathology and atherosclerosis [142]. Distinct miRNA signatures have been reported in cardiovascular disease [143] and these may reasonably be considered as potential novel targets for alcohol and for therapeutic intervention. 

## 10. Conclusions

**Figure 1:**
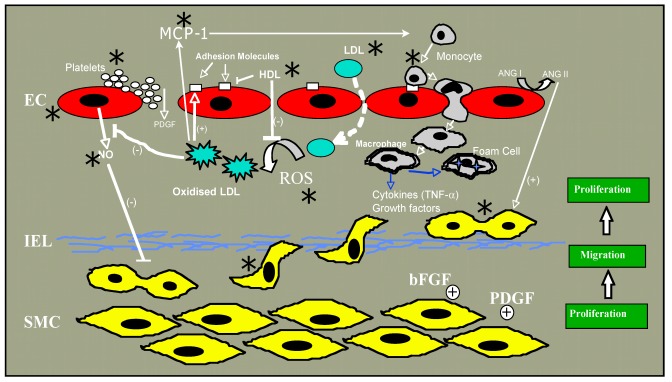
Steps in atherogenesis affected by ethanol are indicated by an asterix (*).

Many epidemiologic studies demonstrate a complex association between alcohol consumption and cardiovascular disease, with frequent low-moderate consumption being protective and chronic abuse or binge drinking being exacerbatory. Research scientists have corroborated these population findings in animal models of atherosclerosis and vascular remodeling, and have attempted to drill down to the cell and molecular signaling mechanisms involved using cultured cells* in vitro *exposed to ethanol. Evidence exists for an alcohol modulation of many of the individual steps deemed crucial in the atherogenic process including effects on lipid levels, inflammation and oxidative stress, as well as the differential effects on endothelial cells and vascular smooth muscle cells reviewed here. Changes in vasoactive substance production, cell migration and growth, enzyme and ion channel activity, and several signaling pathways have been described in response to ethanol exposure. Alcohol’s impact on cardiovascular disease is, therefore, likely the cumulative result of several separate effects (Figure 1). Investigation into the specific molecular mechanisms mediating these responses continues and is warranted. New areas of research interest in the alcohol field include progenitor cell effects, modulation of lipid raft protein trafficking and miRNA gene regulation. Precise knowledge of ethanol’s molecular targets and/or mechanisms of action, together with an understanding of the impact of different patterns of consumption and types of alcoholic beverage on health and disease will help direct healthy drinking behavior and may aid the development of therapeutic agents that can mimic the beneficial effects of alcohol, ideally in the absence of its deleterious and intoxicating effects.
